# New York State Pharmaceutical Association

**Published:** 1881-06

**Authors:** 


### Society Reports.

### NEW YORK STATE PHARMACEUTICAL ASSOCIATION.

The third annual meeting of this association took place in this city, May 18th, 19th and 20th, and brought together such an intelligent body of men that we feel confident that in the effort to elevate and advance the science of medicine, our confreres in Pharmacy will not fail in their duty. The association now numbers over 500, and embraces all of the better educated druggists of the State.

At the opening session the physicians of the city were present in goodly number. The address of welcome on behalf of the medical profession was delivered by Prof. James P. White, M. D., and was substantially as follows:

*Mr. President and Gentlemen of the New York State Pharmaceutical Association :*

I gladly accept the invitation of your committee to extend to you the welcome of the medical profession of the city of Buffalo. In behalf, then, gentlemen, of that noble and generous body of men whom I have on this occasion the honor to represent, I bid you a hearty and cordial fraternal welcome. Pharmacy is a department of the great field of medical science. The medical profession and the pharmaceutists are mutually dependent, each upon the other. These two professional studies and pursuits should therefore recognize their inter-dependence and be warm co-laborers in scientific advancement. My long experience in the practice of the profession of medicine has given me ample opportunity of knowing how greatly the science of medicine has been aided in its "life-saving efforts" by the labors and investigations of pharmacists and chemists. During the present century an apothecary of Einbeck, in Hanover, made the grand discovery of morphia. Quinine has, since I came onto the scene of action, taken the place of crude bark which was formerly administered for the cure of intermitting fever. All the alkaloids have been discovered and introduced by pharmaceutical chemists during the same period. Scheele, an apothecary, enriched medicine by the discovery of many valuable organic bodies, while Davy and Liebig, so justly celebrated as chemists and investigators, commenced their grand careers as apothecary boys The labors of these and many others who might be mentioned, have been of the greatest service to the profession of medicine as well as to mankind. There is indeed a striking contrast between the condition of pharmacy to-day and that which existed half a century ago, when I commenced the study of medicine. Then crude drugs were almost invariably administered, and often their value estimated according to their nauseousness and violence of their action Now, a better understanding of medicines, their isolated principles, and their therapeutical properties, coupled with the advances made in the art and science of pharmacy and chemistry, has enabled us to employ their active principles in concentrated and minute doses, fashioned by skillful hands into tasteless or agreeable simples and compounds Then it was necessary for the doctor to compound and dispense his own medicines, purchasing in bulk the raw material Now the science of medicine has reached to so vast proportions that it is impossible for any one mind to fully master all its departments By a cultivation of the special departments Chemistry, Botany, Physics, Mineralogy, etc., Pharmacy has elevated itself to a certain independence and achieved the right to be ranked as a learned profession We are, I trust, all united upon the common principle that pharmacy can only make intelligent progress under the guidance of educated pharmaceutists and all intelligent physicians are ready to aid you in elevating the standard of education Your chief object should be, then, and I believe it is, to beget and encourage a desire among druggists for a better professional education. Progress is the spirit of this age, and progress in all departments of science and the arts is greatly promoted by the modern idea of association—the attraction of mind with mind—individuals coming together to compare notes; and hence a reason why so large a body of intelligent men are here to day, and I may add why, also, the medical profession extend to you their welcome congratulations. In my opinion every medical school in the country should make suitable provision for instructing the students in pharmacy and to a certain extent in therapeutics, in connection with chemistry and the other studies which all intelligent practical druggists should be required to pursue Whilst I am thoroughly convinced that the interests of the profession are promoted by specialties in its practice, I would insist that no specialist can successfully pursue any department of the profession without being first well grounded in all. Such is the interdependence, that the specialist should be familiar with the whole field before he undertakes the cultivation of a special section thereof. The same general rule will, at no distant day, obtain favorable consideration in the specialty which you practice.

Do not understand me as recommending the dispenser of medicines to attain this knowledge of these properties, in order that he may be able to prescribe them. No; sufficient for him that he skillfully dispenses what his neighboring practitioner has, by his larger experience in diagnosis and more perfect knowledge of pathology. concluded would best cure the patient. The two pursuits should, though intimately wedded, be separate and distinct

To the full accomplishment of your purposes—the proper education of students in your profession, it will be necessary that they undergo a thorough examination and receive from some authorized body a license or certificate of their being suitably qualified for its practice. It is plainly necessary that he who dispenses concentrated remedies, should be so far educated in chemistry and pharmacy as to be able to detect impurities and adulterations in the articles which he is manipulating. Good intentions on his part will be of little avail as a chemical test of purity or impurity, and the druggist must therefore possess such a degree of training and skill as will enable him to know the right and reject the wrong. Indeed the public should demand that he be required to undergo a satisfactory examination and should receive a license from an authorized board as a guarantee that he possesses the requisite qualifications for the exercise of his delicate and responsible trust.

This can only be done by legislative co-operation. It is more important to the public that it should be protected from the danger of incompetency than to your professional security and *esprit de corps*. Obtain, therefore, the passage of such laws as may be necessary to guard against the selling of drugs by unqualified persons Bestow great care upon any bill submitted to the legislature for its adoption. Neither ask too much in the hope that the law alone will make safe dispensers of the active remedies which you are required to prepare for administration, nor should you by timidity or modesty in your demands seek less than will duly protect both you and your patients. Remember, that any defects which may be found by its practical working to exist in this first law, you will be afforded an annual opportunity of correcting by addition or amendment.

Make the beginning at once and leave time and careful observations to perfect its provisions. You will of course be opposed in your efforts to secure legal sanction and support by the non progressive, the mercenary and the ignorant, but you may confidently look for aid from the whole body of intelligent medical practitioners throughout the State, And again I say, obtain such legislative action in behalf of this movement as you can to-day, and rely upon making such modification as may be demonstrated to be important to make it more perefect hereafter. * * *

But, Mr. President, I must not be unmindful of the fact that I am merely invited to say one word and that I am detaining you from important business, and, therefore, wishing you a very successful session, a hospitable reception on the part of all the citizens of Buffalo, and again bidding you. one and all, in the name of the medical profession, a hearty, a cordial welcome, I will close these imperfect suggestions, expressing sincere regret for their imperfections.

The most important business transacted was that relative to the obtainment of the passage of the new pharmacy law. The subject elicited a spirited discussion, a few members from the rural districts being of the opinion that the operation of the law would be injurious to them, while others thought that the provisions of the law were not sufficiently stringent. It was finally resolved (3 voices only dissenting) to heartily endorse the Pharmacy bill as it has passed the Senate and ask its immediate adoption by the Assembly in its entirety, without amendment or alteration.

The following officers were elected for the ensuing year: President, A. B. Huested, M. D., Albany; Secretary, C. W. Holmes, Elmira; Treasurer, C. H. Butler, Oswego.

The fame of our good city for generous hospitality was well sustained by the Buffalo pharmacists, and the visitors were treated to a round of entertainments all carried out with admirable taste and judgment.

